# Computationally designed protein activation

**DOI:** 10.1093/nsr/nwz063

**Published:** 2019-06-03

**Authors:** David Baker

**Affiliations:** 1 Department of Biochemistry, Institute for Protein Design, University of Washington, USA; 2 Howard Hughes Medical Institute, USA

Time-resolved tools for activating proteins of interest in living systems can allow monitoring of the dynamics of biological processes such as signal transduction. Various approaches have been developed to engineer both constitutive and inducible protein activation (e.g. genetic fusion of light switchable proteins). Active-site decaging is another strategy, but the limited number of genetically encoded unnatural amino acids and associated decaging reactions restricts its use to a limited number of protein families.

**Figure 1.**
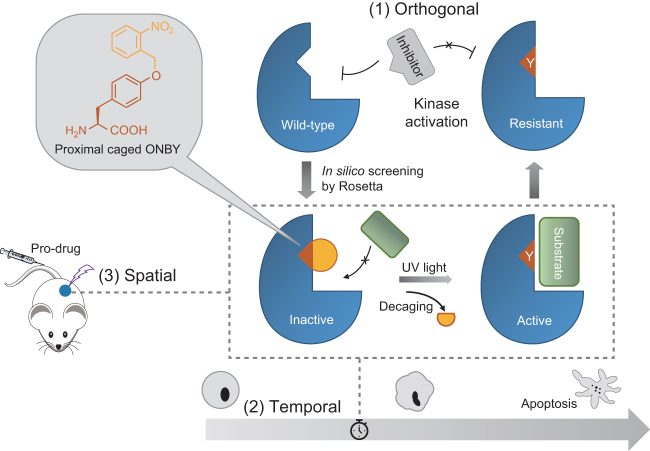
Schematic of CAGE-prox and its three applications. A photo-caged tyrosine (ONBY) is inserted at a proximal position near the active site of protein of interest, which will block the protein's activity. Upon photo decaging, the protein is reactivated. The best position to insert ONBY is recommended by Rosetta *in silico* calculation. CAGE-prox can be applied to (1) construct an orthogonal kinase pathway in which the kinase mutant with the introduced tyrosine confers resistance to the inhibitor; (2) perform temporal profiling of substrates of caspase-3 in the early stage of apoptosis after the protease is activated with time-resolution instantaneously in living cells; (3) achieve spatial activation of a protein-based pro-drug for killing cancers in living animals.

Professor Peng Chen and Professor Chu Wang from the College of Chemistry and Molecular Engineering at Peking University have recently reported a universal protein-activation method in living systems, enabled by computational protein design [1], that they describe as ‘proximal decaging’. The Chen lab has previously developed bioorthogonal cleavage reactions to perform gain-of-function studies of protein families such as kinases by genetically encoded active-site decaging [2,3]. In collaboration with the Wang group, which has deep expertise in bioinformatics and computational modeling for discovering and manipulating functional sites in proteomes, they sought to introduce a photo-caged tyrosine (ONBY) as a universal ‘cage’ at a site near, but not in, the catalytic center of the target protein or enzyme. The caged amino acid blocks protein activity but, upon decaging, the protein activity is immediately unleashed, even in a complex proteome (Fig. 1). The strategy goes beyond traditional active-site-based methods by targeting the substrate/cofactor binding pocket and is in principle capable of controlling the function of almost any protein of interest.

In order to identify suitable sites for ONBY insertion in an unbiased way, the Wang group employed Rosetta [4] to systematically calculate the impact on protein stability and substrate binding of the introduced tyrosine both with and without the caging group—the goal was to identify sites where binding is blocked by the former but not the latter. The team demonstrated the power of the method by caging a variety of enzymes including luciferase, kinase, GTPase, demethylase, caspase and metalloprotease. In all cases, the design calculations succeeded in identifying sites for ONBY incorporation that were experimentally demonstrated to provide effective caging of function and then activation following decaging. Even in the case of nano luciferase, which does not have a defined active site, they were able to successfully cage function (by applying the computational analysis to a Rosetta docking model of the enzyme–substrate complex to identify proximal sites).

The authors showcased their new method—‘Computationally Aided and Genetically Encoded Proximal Decaging’ (CAGE-prox)—in three important applications. Firstly, they constructed an orthogonal MAPK-signaling cascade by creating a MEK1 mutant that is resistant to inhibitors for endogenous wild-type MEK1. Secondly, they activated caspase-3 with CAGE-prox to induce immediate apoptosis and perform a temporal proteomic profiling of its substrates with secondary proteolysis events minimized. Lastly, they explored the potential of *in situ* activating a bacterial lethal factor metalloprotease for killing cancer cells as a protein pro-drug (Fig. 1).

The work by the Chen and Wang groups demonstrates that universal protein activation can be elegantly designed *in silico* and then realized experimentally by cutting-edge chemical biology tools. An exciting next step for the proximal decaging strategy could be to regulate protein interactions with other biomolecules for functional studies in living systems.
